# Interleukin-1 receptor antagonist polymorphisms in women receiving epidural analgesia who develop maternal intrapartum fever: a prospective, multicentre Mendelian randomised study

**DOI:** 10.1016/j.bja.2025.03.037

**Published:** 2025-05-14

**Authors:** EPIFEVER-2 investigators, Amaan Ali, James Noblett, Nusrat Usman, Sarah Wray, Holly Blake, Ana Gutierrez del Arroyo, Tom E.F. Abbott, Salma Begum, Priyanthi Dias, Valentin Weber, Constantinos Papoutsos, Gareth Ackland, Rebecca Black, Ayub Khan, Ben Stretch, Matt Wikner, Rhiannon Wong, Anna Warrington, Kara Bruce-Hickman, Parvesh Verma, Eliana Paola Rodriguez Sierra, Colin Coulter, Faida Al-Maiyah, Alice Barrett, Mira Razzaque, Adam Patrick, Tim Martin, Abhilash Das, Asya Veloso Costa, Chris Palfreeman, Daniel George, Juveria Raja, Lucy Stephenson, Stephanie Kwok, Nadia Quereshi, Arabella Chapman, Jonathan Tsun, Ameesh Patel, Camilla Smith, Aruthy Arumugaum, Meilian Hoe, Charlie Thompson, Ariana Singh, Prasanth Sritharan, Luke Valori, Emma Collins, Natasha Kennedy, Rebecca Longbottom, Michal Rosie Meroz, Tabitha Tanqueray, Lisa Canclini, India Noakes, Rachel Frowd, Thomas Sharp, Ruth Leary, Angela Nicklin, Eftychia Sousi, Ilenia Mazzoli, Pierre Berger, Luca De Freixo, Amrutha Vishwanathan, Paola Eiben, Sally Hoodless, Alison Carey, Angela Pinder, Matt Wilson, Jenny Waspe, Paul Bramley, Vicki Wilson, Anna L. David, Sarah Weist, Olivia Newth, Morenike Folorunsho, Jihana Ali, Yaa Achempong, Miriam Bourke, Derek Brunnen, Jennifer Kim, Kei Mak, Peter Odor, Laura Sarmiento, Sarah Ciechanowicz, Lauren Xuereb Borg, Vikas Tripurneni, Mandeep Phull

**Affiliations:** 1 Royal London Hospital, UK; 2 Whipps Cross Hospital, UK; 3 Homerton Hospital, UK; 4 Sheffield University Hospital, UK; 5 University College London Hospitals NHS Trust/UCL, UK; 6 Barking, Havering and Redbridge University Hospitals, UK

**Keywords:** epidural, fever, genetics, interleukin-1 receptor antagonist, inflammation, intrapartum, Mendelian randomisation

## Abstract

**Background:**

Genetically predicted higher levels of the anti-inflammatory cytokine interleukin-1 receptor antagonist (IL1-Ra) might reduce the risk of developing epidural-related maternal fever, a phenomenon that occurs exclusively in women having epidural analgesia in labour. We hypothesised that in women having epidural analgesia, the absence of specific alleles that lower circulating levels of IL1-Ra would be associated with the development of epidural-related maternal fever, administration of intrapartum antibiotics, or both.

**Methods:**

We prospectively enrolled women ≥18 yr of age receiving epidural analgesia during labour, excluding those with pre-existing fever, antibiotic therapy, or immunodeficiency. Allele scores were constructed from genotyping the C-allele frequency at variants rs6743376 and rs1542176; more copies of each allele independently raise IL-1Ra. The composite primary outcome was maternal intrapartum fever (>38°C) or administration of intrapartum antibiotics after epidural placement. The exposure of interest was the IL1-Ra allele score, comparing 0 (lowest genetically predicted IL-1Ra levels) with ≥1 allele scores. Maternal fever and antibiotic administration were compared in women with 0 or ≥1 allele scores.

**Results:**

Of 624 women genotyped, 155 (24.8%) developed maternal fever or received antibiotics. Fever or antibiotic administration occurred in 19/74 (25.7%) labouring women with an IL-1Ra allele score of 0, compared with 136/550 (24.7%) women with IL-1Ra allele scores ≥1 (odds ratio 1.05, 95% confidence interval 0.60–1.83; *P*=0.89).

**Conclusions:**

In women who receive epidural analgesia during labour, genetically predicted (higher) interleukin-1 receptor antagonist levels do not alter the incidence of maternal intrapartum fever or use of intrapartum antibiotics.

**Clinical trial registration:**

ISRCTN99641204.

**Editor's key points**

• Intrapartum maternal fever affects fetal heart rate and is associated with adverse outcomes. Epidural-related maternal fever occurs in ∼20% of pregnant women who receive epidural labour analgesia.
• The authors hypothesised that in women having epidural analgesia, specific alleles that determine the circulating levels of the anti-inflammatory cytokine interkeukin-1 receptor antagonist (IL1-Ra) are associated with development of epidural-related maternal fever or antibiotic administration.
• Of 624 prospectively enrolled women having epidural analgesia during labour, genetically higher levels of IL1-Ra were not more likely to result in maternal intrapartum fever, intrapartum antibiotic administration, or Caesarean delivery.
• However, intrapartum fever may directly impact obstetric management, increasing the likelihood of Caesarean delivery.

There are ∼600 000 live births in England and Wales each year, of which ∼40% are currently Caesarean deliveries.^1^ Emergent Caesarean delivery during labour is frequently precipitated by fetal tachycardia (>160 beats min^−1^), even in the absence of decelerations or reduced variability.^2^ Intrapartum maternal fever directly affects fetal heart rate and is associated with adverse outcomes for both mother and baby.^3^ Epidural-related maternal fever (ERMF) is one source of maternal intrapartum fever that is observed in ∼20% of pregnant women who receive epidural labour analgesia.^4^ ERMF might develop through a sterile (non-infectious) inflammatory process, promoted by local anaesthetic agents preventing the release of the antipyrogenic cytokine interleukin-1-receptor antagonist (IL-1Ra).^5^ The decrease in plasma IL-1Ra/IL-1β ratio observed in women who receive epidural bupivacaine suggests that IL-1Ra or IL-1β might play a role in ERMF.^5^

The pro-inflammatory effects of IL-1, a master cytokine present in nearly all human cells, are tightly controlled by binding of IL-1 to IL-1Ra.^6^ Genetic variants rs6743376 and rs1542176 are non-correlated and located upstream of *ILRN*, the gene encoding the IL-1 receptor antagonist (IL-1Ra).^7^ These variants are the strongest known genetic determinants of circulating IL-1Ra protein concentrations. For each *IL1RN* C-allele, plasma concentrations of IL-1Ra increase log-fold, with correlative reductions in markers of systemic inflammation, including IL-6 and C-reactive protein.^7^ The construction of a genetic score combining the alleles of rs6743376 and rs1542176 shows that genetically determined increases in IL-1Ra protein concentrations have comparable anti-inflammatory effects to the IL-1R antagonist anakinra.^7^

Using the Mendelian randomisation approach on UK Biobank intrapartum data, we found that genetically predicted higher IL-1Ra was associated with a higher risk of Caesarean delivery after epidural analgesia.^8^ In contrast, women with the lowest genetically predicted IL-1Ra (0 allele score) had similar Caesarean delivery rates, regardless of mode of analgesia. These data can be explained by the hypothesis that neuraxial analgesia, through local anaesthetic agents such as bupivacaine, prevents the release of anti-inflammatory IL-1Ra. This might alter the finely controlled inflammatory processes that help determine the duration of labour, and hence impact the likelihood of obstetric interventions.^5^ Because IL-1Ra is a leaderless protein residing in the cytoplasm awaiting release to counteract acute inflammation, genetically determined levels are not dependent on the rate of mRNA transcription.^6^ Thus, endogenous anti-inflammatory countermeasures within hours of an inflammatory trigger are likely to be critically dependent on the quantity of intracellular IL-1Ra already available for rapid release. Based on our UK Biobank data analysis,^8^ we hypothesised that in women with epidural labour analgesia, maternal intrapartum fever would be less frequent in women with genetically predicted higher IL-1Ra.

## Methods

### Study design

The EPIFEVER-2 study was a prospective observational cohort study using Mendelian randomisation conducted in six UK centres after ethical approval was obtained on January 25, 2021, from the London (Bloomsbury) Research Ethics Committee (20/LO/1213). The study was prospectively registered (ISRCTN99641204) following ethical approval. A protocol paper detailing data collection, design, and analysis has been published.^9^ Participants provided written informed consent. We adhered to STROBE-MR recommendations for strengthening the reporting of observational studies in epidemiology using Mendelian randomisation (Supplementary Table 1).^10^

### Inclusion criteria

This prospective study was designed with strict inclusion and exclusion criteria to minimise the risk of potential confounders in interpreting the relationship between maternal intrapartum fever, clinical outcomes, and allele scores.^9^ Inclusion criteria were as follows: women aged ≥18 yr; singleton or twin pregnancy; and epidural catheter sited for labour analgesia. Exclusion criteria were as follows: unwilling or unable to give consent; inability to understand written or verbal English; known immune or genetic syndromes or mutations, characterised by immunodeficiency; microbiologically proven infection before epidural insertion (including a positive COVID-19 rapid antigen test); established intrapartum fever (maternal temperature ≥38°C); intrauterine death; or antibiotics prescribed or administered with or without a microbiologically proven infection.

### Clinical data collection

**Fig. 1:**
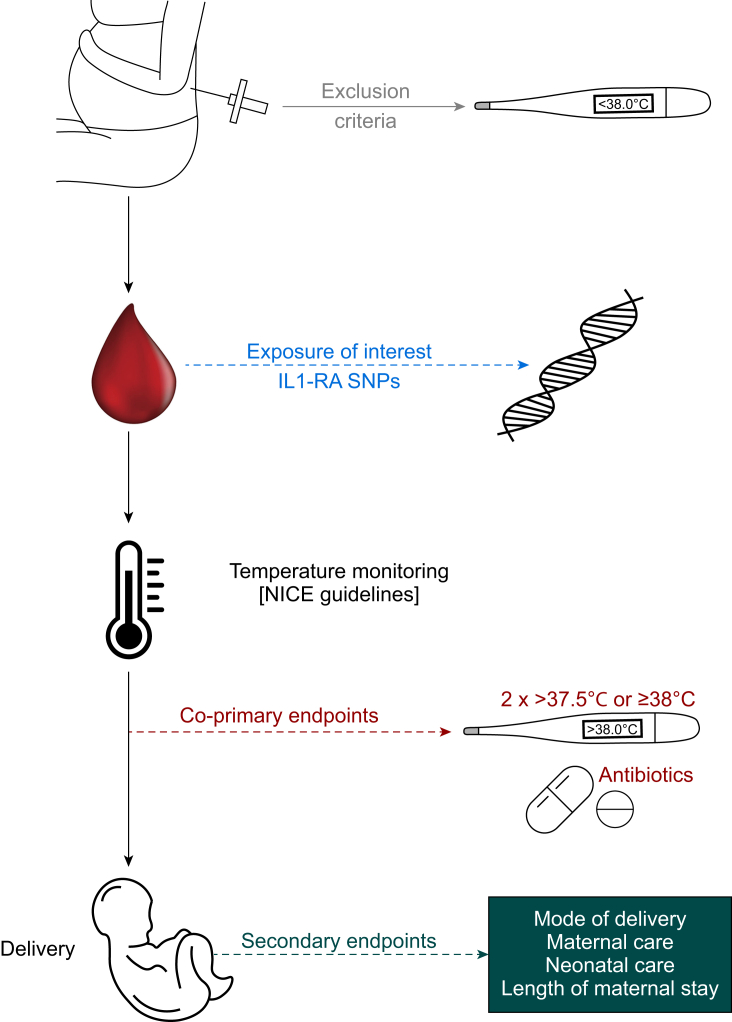
Study protocol. Summary of study procedures and data collection. IL1-Ra, interleukin 1 receptor antagonist; NICE, UK National Institute for Clinical Excellence; SNP, single-nucleotide polymorphism.

The time of epidural insertion and birth were recorded, as were temperature checks guided by routine care guidelines for women receiving epidural analgesia (Fig. 1). Mode of birth, prescription of antibiotics before birth, and length of maternal hospital stay were abstracted from the electronic health record.

### Single-nucleotide polymorphism genotyping

Blood samples were collected within 4 h after epidural catheter insertion in PAXgene Blood DNA tubes (Qiagen, Manchester, UK), the optimal methodology to preserve genomic DNA.^11^ Paxgene tubes were stored at –80° C before analysis. QMUL Genome Centre performed targeted DNA analysis. A batched analysis was undertaken after the end of the study by personnel masked to all study details. Because this project involved a small number of single-nucleotide polymorphisms (SNPs) and a large population, we used Taqman SNP genotyping for the presence or absence of rs6743376 and rs1542176 alleles for IL-1Ra to establish the genetic score. We also explored whether a polymorphism for the NLRP3 (NOD [nucleotide oligomerisation domain]-, LRR [leucine-rich repeat]-, and PYD [pyrin domain]-containing protein 3) inflammasome,^12^ an innate immune signalling complex, is related to ERMF, given that NLRP3 is the key mediator of IL-1 family cytokine production.^13^ We genotyped the NLRP3 intronic variant rs10754555, which is associated with clinically relevant increased systemic inflammation through inflammasome activation and IL-1β production.^12^

### Construction of IL-1Ra allele score

Rather than measure circulating IL1-Ra levels, we adopted the same approach described by the Interleukin 1 Genetics Consortium, which examined the cardiometabolic effects of genetic upregulation of the IL-1Ra using a Mendelian randomisation analysis.^7^ This score met all the criteria required for this approach, chiefly because the two SNPs are uncorrelated and have statistically independent effects on genetically predicted IL-1Ra concentration.^14^ Moreover, both SNPs have similar magnitudes of effect on IL-1Ra concentration, with a linear association between the genetic score and IL-1Ra concentration. **For each** IL1RN **C-allele** inherited, genetically predicted **IL-1Ra** concentrations **increase** by 0.22 standard deviations.^7^ This means that combining both SNPs by constructing a genetic score enhances study power and is biologically relevant. As an exploratory analysis, we also examined the association between NLRP3 intronic variant rs10754555 and the primary outcome.^12^

### Exposure of interest

The exposure of interest was genetically predicted IL-1Ra, comparing 0 (lowest genetic IL-1Ra levels) with ≥1 allele scores.

### Primary outcome

The composite primary outcome was maternal intrapartum fever, defined as maternal temperature >38°C, or prescription of antibiotics during labour after epidural analgesia is commenced before delivery (triggered by the UK Royal College of Obstetrics and Gynaecology guidelines recommending antimicrobial therapy if maternal temperature was either >38°C or >37.5°C for two measurements made at least 1 h apart).^15^ Therefore, in the absence of antibiotics, the primary outcome was met if maternal temperature exceeded 38°C.

### Secondary outcomes

Secondary outcomes were individual components of the co-primary outcome, mode of birth, and clinical care required for the mother (duration of labour and hospital stay, and critical care utilisation) and the baby (Apgar score at 5 min after birth and neonatal critical care utilisation).

### Statistical analysis

For the prespecified primary clinical outcome, the relative incidence of the primary composite outcome was compared between the allele scores 0 and ≥1. For primary and secondary outcomes, we performed a χ^2^ test to establish whether there was independence between (and across) allele scores. For mode of birth, the interaction between the presence or absence of maternal intrapartum fever or antibiotic administration and allele score on Caesarean delivery and vaginal birth rates was explored. For *post hoc* testing, Fisher's exact test (two-tailed) was performed for each χ^2^ test, as statistical simulation studies recommend this as a valuable *post hoc* analysis technique for χ^2^ analysis.^16^ All analyses were performed on an intention-to-treat basis using NCSS 2023 Statistical Software (2023) (NCSS, LLC, Kaysville, UT, USA). Summary statistics of the outcome by group are presented with an estimated treatment effect and corresponding 95% confidence interval (CI) (two-sided *P*-value). For secondary outcomes with low event rates (<20), no statistical analysis was performed. For continuous data, one-way ANOVA was performed, with *post hoc* Tukey–Kramer testing to examine interactions between groups. *P*<0.05 was considered significant.

### Sample size estimate

Based on the IL-1Ra Consortium score^6^ and our analysis of the UK Biobank,^8^ women with an allele score of 0 comprise ∼12% of the population. The overall incidence of maternal intrapartum fever in the first EPIFEVER study was 13.4%,^5^ which we estimated to be very conservative given the lower temperature threshold (37.5°C) now used for early intrapartum antibiotic use, as per UK maternal care guidelines. Before the UK Biobank study analysis, we had surmised that women with an allele score of 0 were more likely to develop maternal intrapartum fever (∼22%). Therefore, we estimated that at least 637 women would be required to detect an absolute difference in the co-primary outcome of ∼13%, assuming 9% incidence of maternal intrapartum fever in women with allele scores ≥1 (α=0.05; 1–β=0.2), an allocation ratio of 7.5:1 and allowing for a dropout rate of 1% after enrolment in labour (two-sided testing). Thus, we estimated 75 women with an allele score of 0 would be compared with 558 women with allele scores ≥1 (PASS 2023 Software (2023); NCSS, LLC).

## Results

### Study participants

**Fig. 2:**
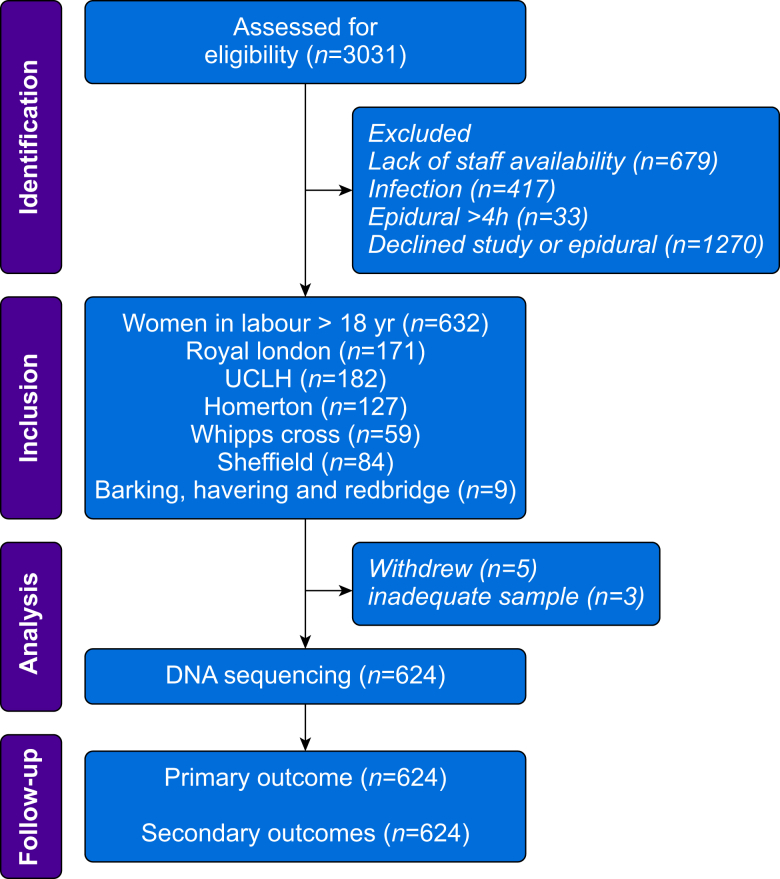
Study flow chart.

Recruitment commenced on April 27, 2021, and was completed on April 25, 2023, in six UK centres. We screened 2399 women during this period, with 632 participating in the study (Fig. 2). Five women withdrew from the study after providing consent to participate; in three women, the DNA samples were of insufficient quality for allele analysis. A large number of women underwent induction of labour, with more than half giving birth for the first time. Opioid analgesia was administered to 109/624 (17.5%) women before epidural analgesia was initiated. Of the 624 participants with complete allele analysis, the mean age was 32 (sd 5) yr with 45% of non-white race (Supplementary Table 1). Sixty-six out of 624 (10.6%) women developed maternal intrapartum fever, of whom 52/66 (78.8%) had blood cultures; 5/52 women had microbial growth reported.

### Distribution of IL-1Ra allele score

Women with an allele score of 0 comprised 11.8% (*n*=74) of the EPIFEVER-2 study population (Supplementary Fig. 1). There were no differences between allele scores for ethnicity or other characteristics (Supplementary Table 2).

### Primary composite outcome: maternal intrapartum fever or antibiotic administration before delivery

**Fig. 3:**
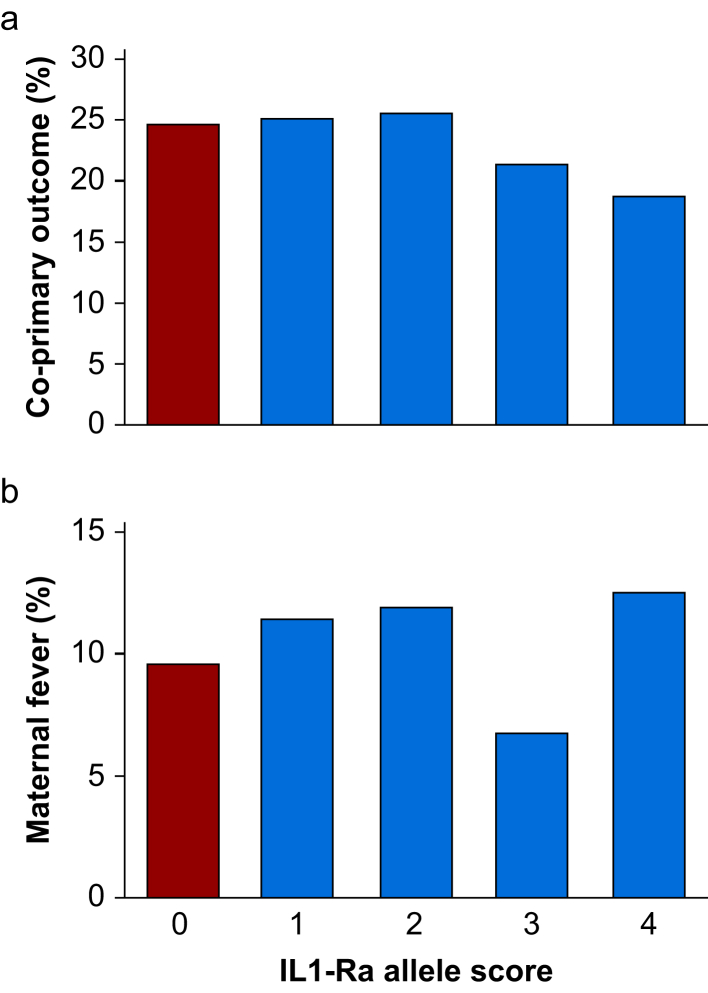
Primary outcome: composite of either intrapartum maternal fever or antibiotic treatment before birth. (a) In total, 19/74 (25.7%) women with an allele score of 0 either developed the co-primary endpoint of intrapartum maternal fever or received antibiotic treatment, compared with 136/550 (24.7%) women with allele scores ≥1 (odds ratio 1.05, 95% CI 0.60–1.83; *P*=0.89). (b) For intrapartum maternal fever alone, 7/74 (9.5%) women with an allele score of 0 developed intrapartum maternal fever, compared with 59/550 (10.7%) women with allele scores ≥1 (odds ratio 0.87, 95% CI 0.38–1.98; *P*=0.84).

For women with an allele score of 0, 19/74 (25.7%) developed either maternal intrapartum fever or received antibiotics (or both), compared with 136/550 (24.7%) women with allele scores ≥1 (odds ratio 1.05, 95% CI 0.60–1.83; *P*=0.89) (Fig. 3a and Supplementary Fig. 2).

### Secondary outcomes

#### Maternal intrapartum fever alone

For maternal intrapartum fever alone, 7/74 (9.5%) women with an allele score of 0 developed fever, compared with 59/550 (10.7%) women with allele scores ≥1 (odds ratio 0.87, 95% CI 0.38–1.98; *P*=0.84) (Fig. 3b).

#### Antibiotic therapy

Intrapartum antibiotics were administered after epidural analgesia was commenced in 20/74 (27.0%) women with an allele score of 0, compared with 133/550 (24.2%) women with allele scores ≥1 (odds ratio 0.87, 95% CI 0.38–1.98; *P*=0.59).

#### Duration of labour

The duration of labour after epidural catheter insertion in women with an allele score of 0 was 644 min (95% CI 565–724 min), compared with 601 min (95% CI 578–631 min) in women with an allele score of 1–4 (*P*=0.31, one-way ANOVA).

#### Mode of delivery

**Fig. 4:**
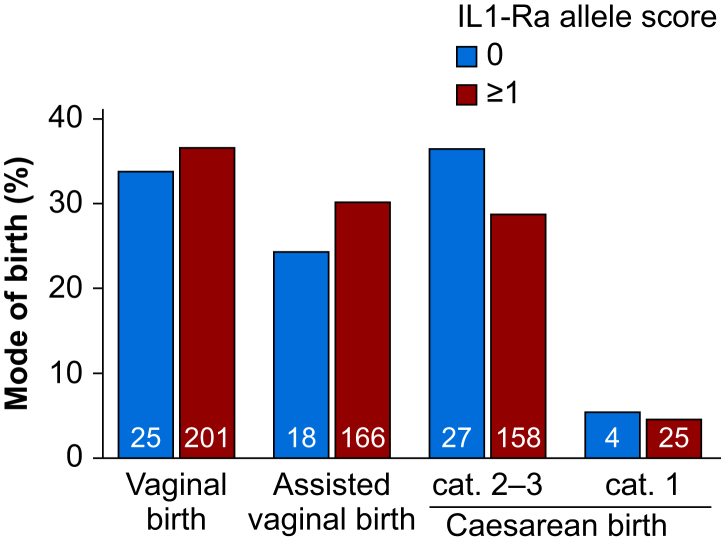
Secondary outcome: mode of birth. Mode of birth in women with 0 or ≥1 allele scores. Cat. 1 and Cat. 2–3 refer to the Caesarean delivery category. The number in each bar refers to the number per allele score grouping.

Caesarean delivery occurred in 31/74 (41.9%) women with an allele score of 0, compared with 183/550 (33.3%) women with allele scores of 1–4 (χ^2^=2.15; *P*=0.143) (Fig. 4 and Supplementary Fig. 3).

#### Neonatal and maternal outcomes

Neonatal and maternal outcomes, including length of maternal hospital stay, did not differ between women with an allele score of 0 and those with allele scores ≥1 (Supplementary Table 3 and Supplementary Figs. 4 and 5). As an exploratory analysis, the NLRP3 intronic variant rs10754555 had no relationship with the primary outcome (χ^2^=2.66; *P*=0.265) (Supplementary Fig. 6).

## Discussion

EPIFEVER-2 has generated the largest prospective dataset detailing the incidence and consequences of maternal intrapartum fever, with strictly defined study entry criteria and exclusion of potential confounding factors. Our primary finding was that, among women who receive neuraxial labour analgesia, the risk of maternal fever is not associated with genetically determined levels of IL-1Ra. Our data also suggest that intrapartum fever in a significant minority of women can directly impact obstetric management.

We designed this prospective study with strict inclusion and exclusion criteria to minimise the risk of potential confounders in interpreting the relationship between maternal intrapartum fever and allele score. Our genetic data, derived using Mendelian randomisation analysis, failed to find a potential causative role for lower IL-1Ra levels in determining the risk of maternal intrapartum fever or antibiotic treatment before birth. The construction of a genetic score for IL-1Ra has enabled Mendelian randomisation studies to examine the causal relation between critical IL-1Ra SNPs and clinical outcomes.^17^ Provided a set of well-characterised assumptions are met, the Mendelian randomisation approach, particularly using allele scores that map to protein levels, provides evidence for a causal relationship between an exposure variable and an outcome.^14^ Because Mendelian randomisation is less likely to be affected by confounding or reverse causation than conventional observational studies,^17^ this approach can offer mechanistic insight into the biological impact of IL-1Ra on outcomes in active labour. Consistent with the UK Biobank, we found similar proportions of women with allele scores to those reported previously.^8^ EPIFEVER-2 extends those findings, as >45% of women were non-white (in contrast to the UK Biobank, where 93% are white British women).

Despite the promise of Mendelian randomisation, where nature effectively randomises individuals, there have been notable discrepancies between the results and pharmacologic RCTs.^17^ three key assumptions need to be met for a Mendelian randomisation study to provide robust insights. First, the genetic variant must be related to the biological parameter of interest. Our laboratory findings suggested a direct link between local anaesthetic (bupivacaine) and IL-1Ra release.^5^ For the second assumption, the current study and our UK Biobank study found no evidence for IL-1Ra correlating with exposure–outcome relationship confounders, including pain,^18^ as evidenced here by similar drug doses across allele scores. The third assumption rests on the ‘no pleiotropy’ rule, which means that the genetic variant affects the outcome only through the risk factor.^14^

A generalisable strength of our study is that the distribution of allele scores in this racially mixed study population was similar to the proportions we reported from the UK Biobank^8^ and those reported by the IL-1Ra Consortium score.^7^ SNPs were processed by batch analysis and reported by personnel blinded to the clinical outcomes. This prospective dataset is highly granular and benefits from tightly controlled inclusion/exclusion criteria. However, the lack of correlative protein data (i.e. circulating IL1β/IL-1Ra) is a limitation. More precise data on when either fever was first detected or antibiotics were administered might have provided further insights. We acknowledge that the secondary outcomes were underpowered. Selection bias in our study is likely, given that women who request labour epidural analgesia often reflect different patient characteristics, such as ethnic group and socioeconomic status, plus other healthcare behaviours, comorbidities, and obstetric history. A further limitation is that the timing and indication of Caesarean delivery were not recorded. Measuring plasma levels of IL-6 and CRP might have offered further insights into maternal fever. It is also possible that the recent use of lipid-formulated RNA vaccines for COVID-19, particularly in this higher-risk pregnant population, might have altered the interleukin 1–interleukin 1 receptor antagonist axis.^19^ We did not record recent COVID-19 vaccination status. The ∼2% dropout rate after intrapartum consent was obtained was higher than expected, but it did not materially affect the main findings of the study.

In summary, for women requesting epidural analgesia during labour, genetically higher levels of IL1-Ra were not more likely to result in maternal intrapartum fever or intrapartum antibiotic administration, or in Caesarean delivery.

## Authors’ contributions

Study conception, design, analysis: GLA.

Sample curation/cataloguing: GLA, AGDA.

First and subsequent drafts of the manuscript: GLA, ALD, AGDA, TEFA, VW, MW, PV, TT, RM, MB, PO.

DNA processing: QMUL Genome Centre (Eva Wozniak, Charles Mein, Ben Jones, Paul Stevens).

## Funding

Project grant from the Obstetric Anaesthetists Association, administered by the National Academy of Academic Anaesthesia; John Snow Vacation scholarship scheme (to AA and FA-M); William Harvey Research Institute Rod Flower vacation scholarship (to VW and AB); Royal College of Anaesthetists/British Oxygen Company research chair grant in anaesthesia, National Institute for Health Research Advanced Fellowship (NIHR300097 to GLA); NIHR Clinical Lectureship and reports grants from Barts Charity, the British Journal of Anaesthesia, and the Royal College of Anaesthetists, during the conduct of the study (to TA); University College London Hospitals Biomedical Research Centre (to ALD).

## Declaration of interest

TEFA and GLA are editorial board members of the *British Journal of Anaesthesia*.

## Appendix 1 EPIFEVER-2 investigators

*Royal London Hospital:* Amaan Ali, James Noblett, Nusrat Usman, Sarah Wray, Holly Blake, Ana Gutierrez del Arroyo, Tom E.F. Abbott, Salma Begum, Priyanthi Dias, Valentin Weber, Constantinos Papoutsos, Gareth Ackland, Rebecca Black, Ayub Khan, Ben Stretch, Matt Wikner, Rhiannon Wong, Anna Warrington, Kara Bruce-Hickman, Parvesh Verma, Eliana Paola Rodriguez Sierra, Colin Coulter, Faida Al-Maiyah, Alice Barrett, Mira Razzaque, Adam Patrick, and Tim Martin.

*Whipps Cross Hospital:* Abhilash Das, Asya Veloso Costa, Chris Palfreeman, Daniel George, Juveria Raja, Lucy Stephenson, Stephanie Kwok, Nadia Quereshi, Arabella Chapman, Jonathan Tsun, Ameesh Patel, Camilla Smith, Aruthy Arumugaum, Meilian Hoe, Charlie Thompson, Ariana Singh, Prasanth Sritharan, Luke Valori, Emma Collins, Natasha Kennedy, and Rebecca Longbottom.

*Homerton Hospital:* Michal Rosie Meroz, Tabitha Tanqueray, Rebecca Longbottom, Lisa Canclini, India Noakes, Rachel Frowd, Natasha Kennedy, Thomas Sharp, Ruth Leary, Angela Nicklin, Eftychia Sousi, Ilenia Mazzoli, Pierre Berger, Anna Warrington, Luca De Freixo, Amrutha Vishwanathan, and Paola Eiben.

*Sheffield University Hospital:* Sally Hoodless, Alison Carey, Angela Pinder, Matt Wilson, Jenny Waspe, Paul Bramley, and Vicki Wilson.

*University College London Hospitals NHS Trust/UCL:* Anna L. David, Sarah Weist, Olivia Newth, Morenike Folorunsho, Jihana Ali, Yaa Achempong, Miriam Bourke, Derek Brunnen, Emma Collins, Jennifer Kim, Kei Mak, Peter Odor, Laura Sarmiento, and Sarah Ciechanowicz, and Lauren Xuereb Borg.

*Barking, Havering and Redbridge University Hospitals:* Vikas Tripurneni, Mandeep Phull, and Ayub Khan.

Genome Centre, Faculty of Medicine and Dentistry, Queen Mary University of London, Blizard Institute (Eva Wozniak, Charles Mein, Ben Jones, Paul Stevens).
